# Single-Cell Data and Weighted Correlation Network Analysis Revealed the Regulatory Mechanisms of Macrophages in Carotid Plaques

**DOI:** 10.1155/jimr/9987367

**Published:** 2025-07-21

**Authors:** Yakun Ding, Xiaoyang Niu, Peng Guo, Bing Wang

**Affiliations:** Department of Vascular Surgery, The Fifth Affiliated Hospital of Zhengzhou University, Zhengzhou, China

**Keywords:** carotid plaque, immune, *NF-κB*, single-cell RNA sequencing, weighted correlation network analysis

## Abstract

**Background:** Macrophages play a critical role in carotid plaque. Understanding the mechanisms of carotid plaque formation based on macrophage heterogeneity could provide valuable insights for clinical intervention.

**Methods:** Single-cell transcriptome and bulk RNA-seq data of carotid plaque were obtained from public databases. Weighted gene correlation network analysis (WGCNA) identified gene modules linked to unstable plaques. Macrophage marker genes were intersected with module genes of WGCNA, followed by using randomForest and LASSO regression to pinpoint key genes. Quantitative real-time PCR (qRT-PCR) and Western blot were used to verify the regulation of key genes at the cellular level. The correlation between the key genes and inflammatory phenotypes was examined by single-sample gene set enrichment analysis (ssGSEA).

**Results:** Single-cell clustering revealed major cellular subpopulations, with elevated macrophage infiltration in carotid plaque. Six key macrophage-associated genes (*ADPGK, ATP6V1F, CX3CR1, MYO9B, RNF135*, and *SLC7A8*) were discovered. The qRT-PCR results demonstrated upregulation of *ADPGK, ATP6V1F*, and *RNF135* genes in vascular smooth muscle cells (VSMCs) treated with oxidized low-density lipoprotein (ox-LDL), except for *CX3CR1*, which was downregulated. Protein expression results showed that expressions of *ADPGK, ATP6V1F, RNF135*, and *SLC7A8* were significantly elevated in the ox-LDL-VSMC group. In addition, most of the immune cells showed significant differences between the unstable arterial plaque group and the control group.

**Conclusion:** This study discovered potential biomarkers that affected carotid plaque progression and macrophage regulation at the single-cell level, and examined their regulatory roles in immune regulation, programed cell death (PCD), and inflammatory factor modulation.

## 1. Introduction

Carotid plaque, a localized atherosclerotic lesion at the common carotid artery bifurcation, is a major contributor to stroke. Atherosclerosis arises from long-term lipid metabolism disorder and is characterized by the accumulation of lipids and complex sugars at the intima-media of the arteries, leading to localized thickening of the intima [1]. On this basis, connective tissue accumulates to form fibrous plaques; consequently, calcium deposits may occur and cause progressive metamorphosis and calcification. As plaques enlarge, the trophoblastic vessels of the adventitia penetrate the media into the thickened intima to form a microvascular network to supply blood to the plaque, resulting in ischemic necrosis of the blood-supplying tissues [2]. Current treatments for carotid plaques include intensive pharmacologic therapy and surgical resection with carotid stenting. Statins are at the core of intensive pharmacologic therapy to reverse or reduce plaque, but they often fail to achieve the target level of low-density lipoprotein (LDL) cholesterol and may cause liver dysfunction [3, 4]. Also, surgery carries risks of perioperative stroke and mortality. Therefore, it is urgent to develop effective therapeutic strategies to slow down the progression of carotid plaque.

Macrophages are central to carotid plaque pathogenesis. During atherosclerosis, LDL cholesterol accumulates within the vessel wall, leading to impaired endothelial cell function, and macrophages transform into foam cells by recognizing and phagocytosing these oxidized LDL (ox-LDL) particles, resulting in the formation of lipid-enriched plaques in the vessel wall [5–7]. These foam cells accumulate and release inflammatory factors to promote localized inflammatory response, further triggering cellular and tissue damage. Plaque may become unstable if it continues to grow and inflammation persists, which will increase the risk of rupture and cause acute cardiovascular events, such as stroke or myocardial infarction [8]. The M1/M2 macrophages play a crucial role in tumor progression. Generally, M1 macrophages exert antitumor effects and M2 macrophages promote tumorigenesis [9]. M1 macrophages emerge in an inflammatory environment dominated by toll-like receptor (TLR) and interferon signaling, whereas M2 macrophages are present in an environment dominated by TH2 response [10]. Study found that the stimulation of macrophage polarization by advanced glycation end products (AGEs) can lead to a rapid development of atherosclerosis [11]. Immunotherapy, a commonly used cancer treatment [12], can also influence atherosclerotic plaques. It has been reported that the volume of noncalcified plaques is significantly increased and the plaques are prone to rupture in patients with lung cancer and melanoma treated with immune checkpoint inhibitors (ICIs), suggesting a higher risk of cardiovascular events [13]. Nonetheless, to better improve the diagnosis and treatment of atherosclerosis, the specific mechanisms of macrophages in carotid plaques should be further elucidated.

This study investigated macrophage-mediated immune pathway activation in unstable carotid plaques at the single-cell level. We identified unstable plaque-associated gene modules through weighted gene correlation network analysis (WGCNA) and employed machine learning algorithms to screen key genes for diagnostic model construction. The diagnostic performance of the model was verified as well. Finally, this study discovered a close connection between the key genes and inflammation-related pathways. Our findings highlighted the diagnostic significance of macrophage-related marker genes in carotid plaques, and revealed the correlation between the model and inflammation-related pathways, providing novel insights for understanding the molecular mechanisms of arterial plaques and guiding clinical interventions. And this is the first study to integrate single-cell RNA-seq with WGCNA to identify macrophage-specific hub genes (*RNF135, SLC7A8*) in carotid plaques.

## 2. Materials and Methods

### 2.1. Acquisition and Preprocessing of the scRNA-Seq Data Related to Arterial Plaque

GSE120521 and GSE41571 datasets containing the bulk RNA-seq data of arterial plaque samples were obtained from the Gene Expression Omnibus (GEO, https://www.ncbi.nlm.nih.gov/geo/). The probes were converted to Symbol according to the annotation file. GSE120521 is a single-cell RNA-seq dataset that stores four unstable plaque samples and four stable plaque samples. A total of 11 control samples (five ruptured plaque samples and six stable plaque samples) were collected from GSE41571.

The scRNA-seq data were preprocessed using the Read10X function of the Seurat package to collect qualified cells with fewer than 10% of mitochondrial genes and gene numbers between 200 and 5000 [14]. The data matrix was normalized by the NormalizeData function, followed by the identification of the top 2000 highly variable genes with the FindVariableFeature function. After that, the 2000 genes were scaled by the ScaleData function before using the RunPCA function to perform linear dimensionality reduction with principal component analysis (PCA) [15–17]. Next, cells were clustered employing the FindNeighbors and FindCluster (original default Louvain algorithm) functions. Finally, the t-distributed stochastic neighbor embedding (tSNE) method was applied for nonlinear dimensionality reduction and visualization of the results.

### 2.2. WGCNA

In this study, WGCNA was utilized to identify gene modules linked to unstable plaques. Initial data preprocessing excluded genes whose expression was 0 in 80% of the samples, while retaining the top 25% most variable genes based on their median absolute deviation (MAD). A sample clustering tree was developed using hierarchical clustering. The similarity matrix was constructed using the Pearson's correlation test and then converted into an adjacency matrix to approximate scale-free topology using power function (*β*), which was determined when the correlation between average connectivity of modules (*k*) and p(*k*) reached 0.85. Subsequently, the adjacency matrix was transformed into a topology overlap matrix (TOM). Finally, hierarchical clustering was conducted to merge highly similar gene modules (the minimum gene number in a module was 50) [18, 19].

### 2.3. Key Genes Screened by Machine Learning Algorithms

The macrophage-specific genes with high expressions were intersected with the genes within the black module, and the resulting genes were compressed by the random forest method and LASSO analysis. The randomForest function in the R package was used to create a random forest model. The number of variables for binary trees in the specified nodes in the model (mtry) was determined based on the out-of-bag (OOB) rate. Next, the number of decision trees (ntree) was determined according to the correlation between the model error and decision tree number. Using these parameters, we developed a random forest model and identified the top 30 genes based on MeanDecreaseAccuracy and MeanDecreasGini graphing. Key markers were selected using the LASSO regression method with 10-fold cross-validation using the R package glmnet. Finally, final genes were obtained by taking the intersection of genes identified by both random forest and LASSO analyses.

### 2.4. Diagnostic Model Construction for Carotid Artery Plaque

In order to analyze the diagnostic performance of key genes, this study analyzed the differential expression of the key genes in carotid plaque samples in different states and plotted the ROC curves.

### 2.5. Single-Sample Gene Set Enrichment Analysis (ssGSEA)

Using the R package GSVA, ssGSEA enrichment scores for immune cell regulation, cell death types, and inflammatory pathways in each sample were calculated, and the correlations between these scores and key genes were examined.

### 2.6. Cell Culture and Treatment

Human aortic vascular smooth muscle cell line (HA-VSMCs) ordered from American Type Culture Collection (ATCC, Manassas, Virginia, United States of America) was cultured in F-12K medium supplemented with 10% FBS (21127030, Thermo Fisher Scientific, Waltham, Massachusetts, USA). To model the pathogenesis of carotid atherosclerosis, HA-VSMCs were treated with ox-LDL [20] (Cat. No. YB-002, Yiyuan Biotechnology, Guangzhou, China) for 24 h. The VSMCs were divided into control and ox-LDL (80 μg/ml, 24 h) groups. The cells were maintained at 37°C under the condition of saturated humidity with 5% CO_2_ [21].

### 2.7. Quantitative Real-Time PCR (qRT-PCR)

Total RNA from cells was extracted by TRIzol RNA extraction reagent (155960261, ThermoScientific, Waltham, Massachusetts, United States of America), and then reverse-transcribed to cDNA using the Qiagen One-Step RT-PCR kit (210212, Qiagen GmbH, Hilden, Germany). Next, qRT-PCR was carried out to detect the expressions of the key genes. Amplification was performed on an ABI 7500 system (4351106, Thermo Fisher Scientific, Waltham, Massachusetts, United States of America) using SYBR Green (D7260, Beyotime, Shanghai, China). Relative mRNA expressions were normalized to the level of GAPDH and calculated with the 2^−ΔΔct^ method. The primer sequences were in Supporting Infromation 1: Table S1.

### 2.8. Western Blot

Protein extracts of cells were prepared using RIPA cell lysis buffer (P0013B, Beyotime, Shanghai, China) and their concentrations were tested using the BCA Protein Assay Kit (P0011, Beyotime, Shanghai, China). Protein extracts (20 μg) were separated in 10% SDS-PAGE separation gel (P0012A, Beyotime, Shanghai, China) and transferred to a polyvinylidene difluoride (PVDF) microporous membrane (FFP36, Beyotime, Shanghai, China). The membranes were placed in Tris-buffered saline (TBS) (10X) (ST661, Beyotime, Shanghai, China) containing 0.1% Tween-20 (ST825, Beyotime, Shanghai, China) and 5% skimmed milk for 2 h at room temperature and then blocked with the appropriate primary antibody (*CX3CR1*: ab308613, *ADPGK*: ab228633, ATP6V1F: ab190789, *RNF135*: ab229959, *SLC7A8*: ab273674, and GAPDH: ab8245, Abcam, Cambridge, UK; MYO9B: NB100-41092, Novus Biologicals, Centennial, CO, USA) overnight at 4°C. The membranes were then incubated with relevant hydrogen peroxide-conjugated secondary antibodies, goat anti-rabbit IgG (ab205718, 1:5000, Abcam, Cambridge, UK) or goat anti-mouse IgG (ab205719, 1:5000, Abcam, Cambridge, UK), for 2 h at room temperature. Then the color was developed with ECL coloring reagent (P0018FS, Beyotime, Shanghai, China) and exposed to the Tanon 4600 automatic chemiluminescence analysis system (Tanon, Shanghai, China). ImageJ software (version 1.45 s, National Institutes of Health, Bethesda, Massachusetts, USA) was applied to calculate relative gray values.

### 2.9. Statistical Analysis

All statistical analyses were conducted in R language (version 3.6.0). The Wilcoxon rank-sum test was employed to calculate the differences between the two groups of continuous variables. For experimental data, two-way Analysis of Variance (ANOVA) was conducted, followed by Bonferroni's multiple comparisons test, in GraphPad Prism software (version 8.0.2). The Spearman method was used for correlation analysis, and *p* < 0.05 was considered to be statistically different, ns means not significant.

## 3. Results

### 3.1. Single-Cell Landscape of Carotid Plaques

**Figure 1:**
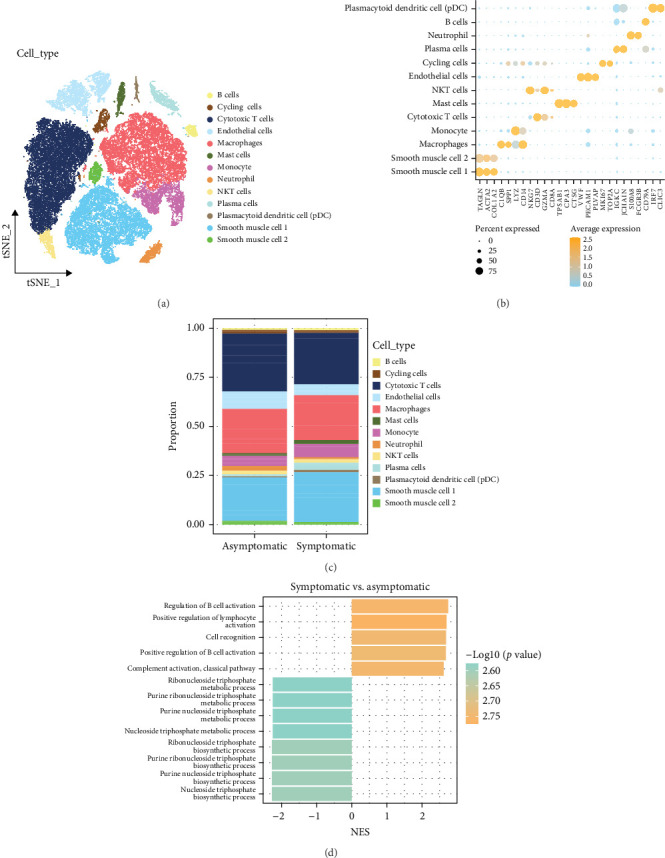
Single cell atlas of carotid plaque tissue. (A) Plots of single-cell landscape in carotid plagues after preprocessing. (B) Expression of marker genes in carotid plaque samples by cell type. (C) Infiltration ratios of each cell subpopulation between carotid plaque symptomatic and asymptomatic samples. (D) GSEA analysis between carotid plaque symptomatic and asymptomatic samples, where normalized enrichment score (NES) > 0 is the pathway enriched in symptomatic samples and NES < 0 is the result for asymptomatic samples.

In this study, 47,183 cells were obtained after preprocessing the single-cell data of carotid plaque from GSE253903 dataset. These cells were classified into 13 cell types, namely, B cells, cycling cells, cytotoxic T cells, endothelial cells, macrophages, mast cells, monocyte, neutrophil, NKT cells, plasma cells, plasmacytoid dendritic cells, smooth muscle cell 1, and smooth muscle cell 2 (Figure 1A,B). Next, we compared the infiltration of each cell type between symptomatic and asymptomatic samples (Figure 1C and Supporting Information 2: Table S2). Since macrophages play an important role in carotid plaques, we extracted macrophage subpopulations and performed GSEA analysis on symptomatic and asymptomatic samples. The results showed that immune cell-associated pathways, including macrophages, were activated in symptomatic samples (Figure 1D).

### 3.2. Unstable Plaque-Associated Gene Modules Identified by WGCNA

**Figure 2:**
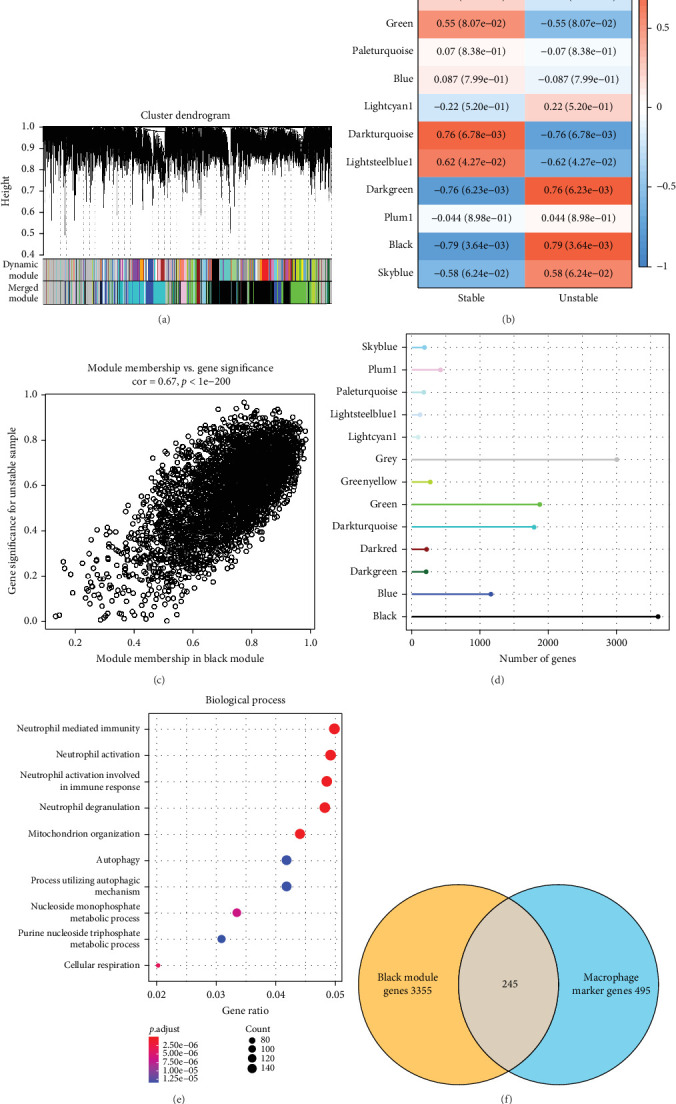
WGCNA analysis of carotid artery plaque samples. (A) Gene dendrogram based on dissimilarity metric (1-TOM) clustering. (B) Correlation of module eigenvectors of each module with unstable patches. (C) Scatter diagram for module membership vs. gene significance for unstable sample in the black module. (D) The number of each cluster in WGCNA. (E) Biological process enrichment results for the black module. (F) Venn diagram for obtaining intersecting genes.

To screen genes associated with unstable carotid artery plaque, we utilized the R package WGCNA to identify gene modules related to unstable carotid artery plaque in GSE41571 dataset. The samples were clustered to classify coexpression modules; a soft threshold of β = 30 was chosen to ensure a scale-free network (Supporting Information 3: Figure S1 and Supporting Information 4: Table S3). Next, gene modules were identified by hierarchical clustering, and 13 coexpression modules were obtained after merging, among which the black module was closely linked to unstable carotid artery plaque (Figure 2A–D). GO enrichment analysis was performed to explore the biological processes and related signaling pathways involved in the black module genes. It was found that these genes were significantly enriched to neutrophil-mediated and neutrophil-activated pathways (Figure 2E).

**Figure 3:**
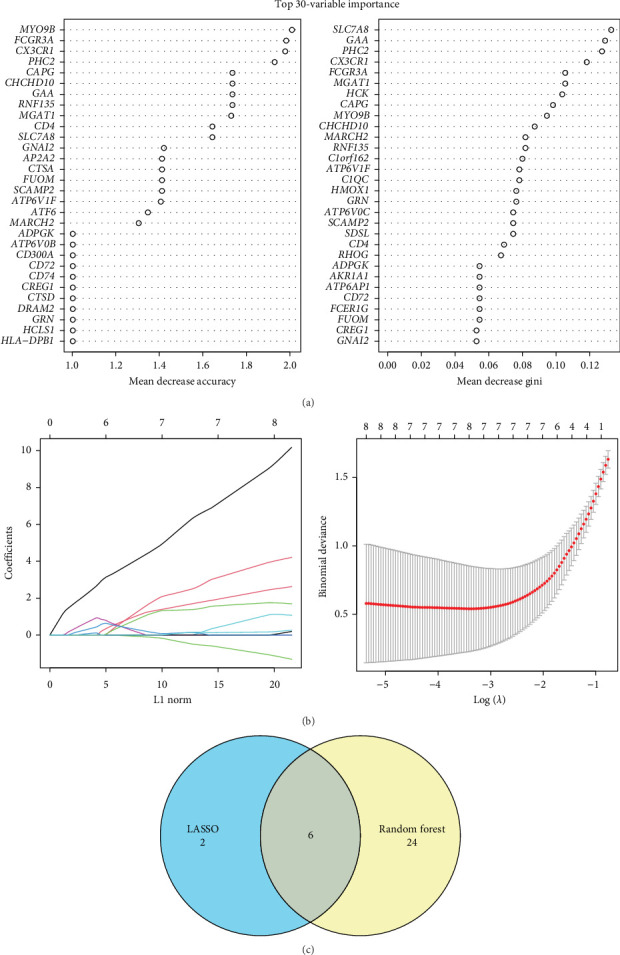
Machine learning screening of key genes related to macrophage regulation. (A) Top 30 contributing markers filtered by random forest model. (B) Changes in coefficients with λ-penalty for different genes and LASSO regression model coefficients fit. (C) Venn diagrams of genes obtained from LASSO analysis and random forest analysis.

Next, genes in the intersection between macrophage-specific high-expressed genes and black module genes (Figure 2F) were further selected using a random forest approach. The top 30 markers were ranked by MeanDecreaseAccuracy and MeanDecreaseGini, respectively (Figure 3A). The range of candidate genes was further reduced by LASSO regression analysis (Figure 3B). As the minimum binomial deviance corresponding to λ was 8, the key markers were determined to be eight. Key genes were obtained by taking the intersection of the genes screened by both the LASSO method and the random forest method. Six key genes (*ADPGK, ATP6V1F, CX3CR1, MYO9B, RNF135*, and *SLC7A8*) related to unstable plaques and macrophages were finally determined (Figure 3C).

### 3.3. Diagnostic Performance and Validation of the Key Genes Linked to Unstable Plaques and Macrophage Regulation

**Figure 4:**
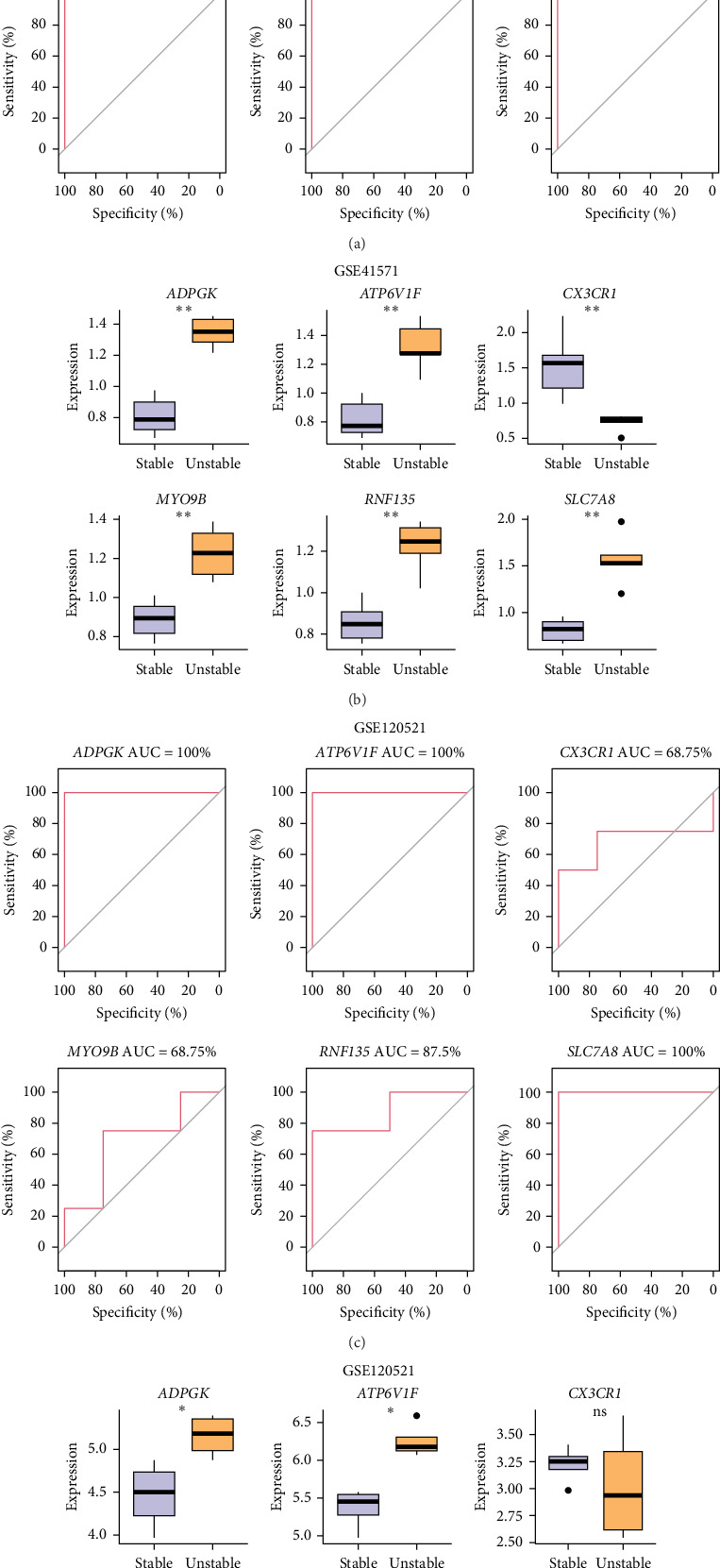
Diagnostic model construction of macrophage and carotid artery plaque-related genes. (A, B) ROC curves and relative expression levels of biomarker diagnostic models in GSE41571. (C, D) ROC curves and relative expression levels of biomarker diagnostic models in GSE120521. *⁣*^*∗*^*p* < 0.05 and *⁣*^*∗∗*^*p* < 0.01. ns means *p* > 0.05.

We evaluated the diagnostic potential of the six identified key genes by plotting ROC curves in the training set GSE41571. All the genes showed an AUC value higher than 0.7, confirming its excellent diagnostic performance (Figure 4A). Differential expression analysis on the six key genes in carotid plaques in different states showed that apart from *CX3CR1*, the relative expressions of the other five genes were remarkably higher in the unstable plaque group than in the stable plaque group (Figure 4B). Meanwhile, with the GSE120521 dataset as a validation set, it was observed that these genes also had a better diagnostic efficacy, and the gene expression trend was consistent with the training set (Figure 4C,D).

### 3.4. Aberrant Expression of Key Genes in Ox-LDL-VSMCs as a Potential Factor Influencing Carotid Plaques

**Figure 5:**
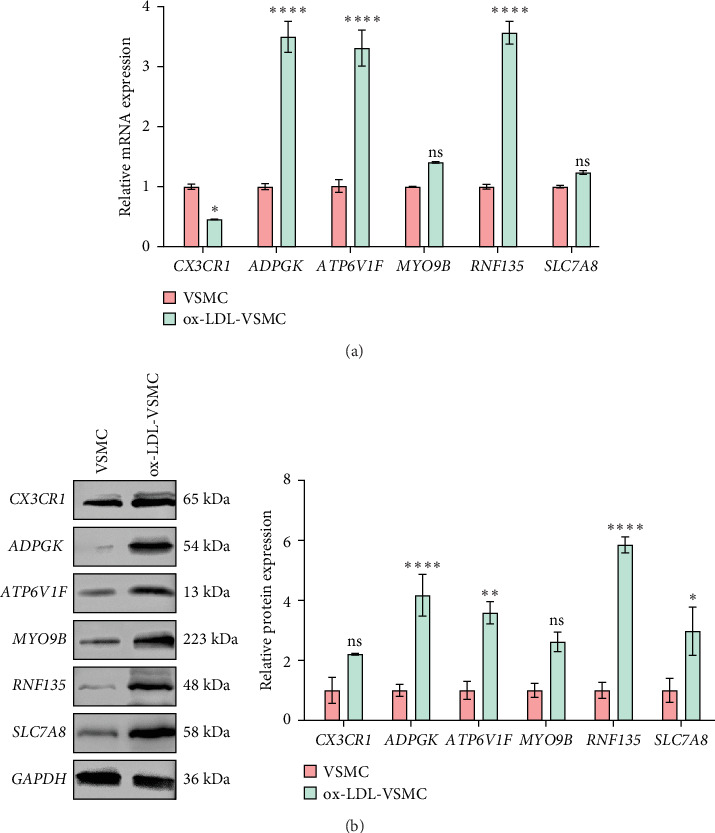
Key genes were verified in vitro. (A) Relative mRNA expression levels of the key genes using qRT-PCR. (B) Western blot analysis of key genes. *⁣*^*∗*^*p* < 0.05, *⁣*^*∗∗*^*p* < 0.01, and *⁣*^*∗∗∗∗*^*p* < 0.0001. ns means *p* > 0.05.

To verify the regulatory effects of the six key genes on carotid artery plaque, their expressions in VSMCs treated by ox-LDL were detected by applying molecular assays. The results showed that *ADPGK, ATP6V1F*, and *RNF135* were significantly upregulated in the ox-LDL group, while the *CX3CR1* mRNA level was notably downregulated (Figure 5A). Protein expression results showed that the expression of *CX3CR1* and *MYO9B* was not significantly different between the ox-LDL-VSMC group and the VSMC group, whereas the expression of *ADPGK, ATP6V1F, RNF135*, and *SLC7A8* was significantly elevated in the ox-LDL-VSMC group (Figure 5B). This result correlates with the multilevel complexity of gene expression regulation, but the relevance of *ADPGK, ATP6V1F, RNF135*, and *SLC7A8* to carotid plaque has been further confirmed.

### 3.5. Correlation of Key Genes With Immune Cell Infiltration and Activation of Inflammatory Pathways

**Figure 6:**
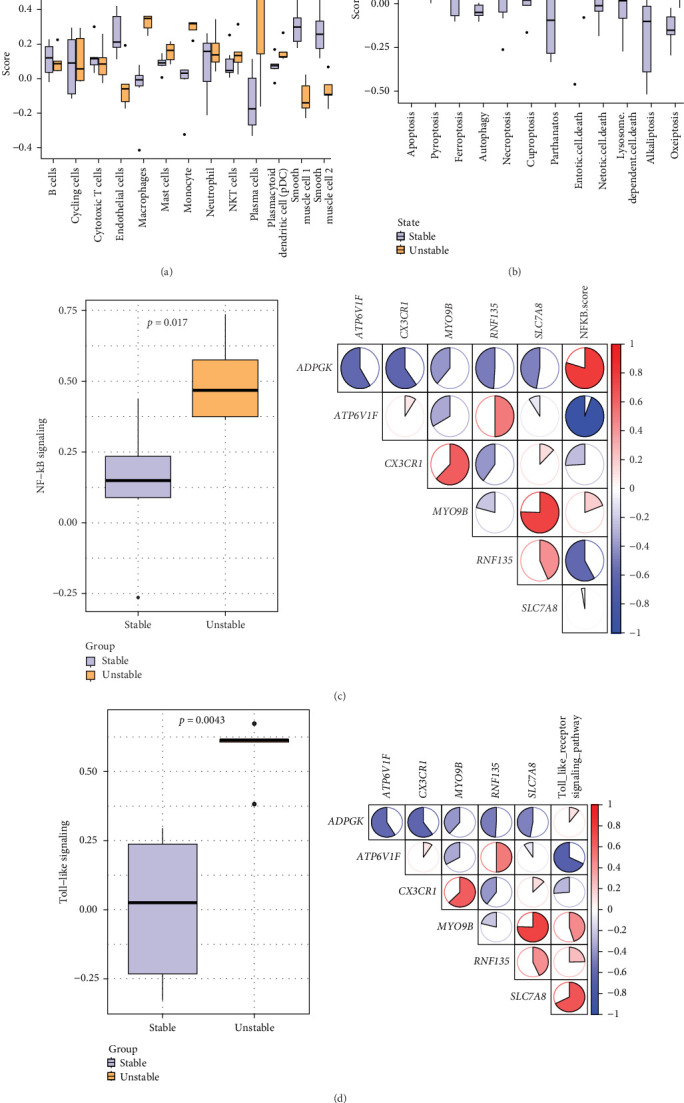
Correlation of key gene with immune cell infiltration, type of programed cell death and inflammatory pathways. (A) Differences in immune cell scores of carotid plaques of different states in GSE41571. (B) Differences in programed death scores of carotid plaques of different states in GSE41571. (C) Comparison of *NF-κB* signaling pathway scores in different states of carotid plaques and correlation analysis with key genes in GSE41571. (D) Evaluation of toll-like signaling pathway scores in diverse carotid plaque conditions and correlation with key genes. *⁣*^*∗*^*p* < 0.05 and *⁣*^*∗∗*^*p* < 0.01. ns means *p* > 0.05.

We used the ssGSEA method in the GSVA package to calculate cell-specific scores of each sample in the GSE41571 dataset and to compare the differences in these scores in carotid plaques in different states. It was found that the infiltration of smooth muscle cells, endothelial cells, macrophages, monocytes, plasma cells, and some other immune cells in the unstable plaque group was significantly different than that in the stable group (Figure 6A). In addition, the scores related to PCD were higher in unstable plaques, indicating that PCD functioned crucially in atherosclerotic plaque formation and progression (Figure 6B). Finally, we investigated the correlation between inflammatory signaling pathways associated with atherosclerosis and the key genes, with a particular focus on the *NF-κB* signaling pathway and the TLR signaling pathway due to their crucial roles in inflammation and immune responses. The results showed significant differences in the activities of *NF-κB* and toll-like signaling pathways between the stable carotid plaque group and unstable plaque group, with the unstable plaque group showing higher activities (Figure 6C,D).

## 4. Discussion

Existing studies reported that macrophage polarization plays an important role in carotid plaque formation, but the underlying mechanism remains largely unclear. Hence, elucidating the mechanisms based on macrophage-associated marker genes in carotid plaque formation is important for improving the prevention and clinical treatment of carotid plaque. The present study applied single-cell transcriptome analysis to examine the regulatory role of macrophages in carotid plaque formation. Through integrated WGCNA and COX regression analyses, we discovered marker genes linked to carotid plaques and macrophages and revealed their potential relevance in the regulation of immunomodulation, cell death mode, and release of inflammatory factors in carotid plaques.

In this study, single-cell clustering combined with WGCNA and multiple COX regression analyses determined *ADPGK, ATP6V1F, CX3CR1, MYO9B, RNF135*, and *SLC7A8* as the molecular markers linked to carotid artery plaque and macrophage regulation. *ADPGK* acts as an inhibitor of pyruvate dehydrogenase kinase and can inhibit platelet aggregation and arterial thrombosis [22]. Gene variants in the coagulation system have been found to be involved in carotid plaques [23], implying that elevated *ADPGK* is related to carotid plaque formation. *ATP6V1F* is often regarded as an immunotherapeutic target for cancers and can regulate cancer progression by activating immune-related pathways [24]. *ATP6V1F* is a part of the central stalk of ATPase (V-ATPase) [25]. V-ATPase mediates organelle acidification, and *ATP6V1F* may therefore, influence carotid plaque development by affecting V-ATPase [26]. *CX3CR1* is a chemokine and a typical regulator of atherosclerosis development [27]. Chemokines, including *CX3CR1*, could regulate atherosclerosis through mediating the targeting of Stabilin factor by immune cells, such as monocytes and macrophages [28]. *MYO9B*, a risk factor for coronary artery diseases, can enhance vascular diseases through modulating vascular cell motility [29]. *RNF135*, a ubiquitin ligase, is involved in intracellular protein degradation. Recent studies revealed that *RNF135* is highly expressed in patients with atherosclerosis and mediates cardiomyocyte survival and repair under ischemic stress, showing its involvement in cardiovascular diseases [30]. Since small, nonstenotic carotid plaques represent a unique phenotypic feature of subclinical atherosclerosis [31], the association between elevated levels of *RNF135* and the progression of carotid plaques requires further investigation. *SLC7A8* is an amino acid transporter protein responsible for the transport of neutral amino acids. The study reported that *SLC7A8* may regulate cardiovascular diseases by influencing cellular functions through energy metabolism in cardiomyocytes [32]. In addition, leucine and isoleucine transported by *SLC7A8* are important for maintaining normal metabolic functions of immune cells and regulating cellular activation through pathways, such as mTOR, thereby influencing the progression of cardiovascular diseases, including atherosclerosis [33]. The above findings indicated that these marker genes could regulate cardiovascular diseases and disease progression through modulating immune cell activity, which was consistent with the results of the present study that macrophage activity and carotid plaque formation were inextricably linked to the mediation of these genes.

Enrichment analysis of the module genes showed that the genes were mainly enriched in pathways such as neutrophil-mediated immunity. Since the relationship between neutrophils and plaque development is well established [34], the current result also supported the correlation between the genes discovered in this study and carotid plaque. In addition, enrichment analysis showed that the marker genes associated with macrophage regulation and carotid plaque progression were significantly enriched in inflammation-related signaling pathways, such as TLRs, *NF-κB*, etc. TLRs are a family of type-recognition receptors that function crucially in autoimmune responses and serve as a bridge between the development of cardiovascular diseases and the immune system [35]. It has been shown that TLRs trigger innate immune responses by recognizing danger-associated molecular patterns (DAMPs) and pathogen-associated molecular patterns (PAMPs), which can modulate many acute and chronic inflammatory diseases [36]. During atherosclerosis, TLRs influence the onset and progression of the disease through initiating endothelial dysfunction, the interaction of various immune cells, and activation of many other inflammatory pathways [20]. *NF-κB* is widely involved in the cellular immune response and inflammatory response. When macrophages, endothelial cells, and smooth muscle cells are stimulated, the *NF-κB* pathway is activated through the induction of IL-6, TNF-α, and some other inflammatory factors to promote the migration of monocytes to the vessel wall, thereby causing the formation of atherosclerotic lesions [37]. This also indicated that the *NF-κB* pathway is inextricably linked to macrophage activity. In addition, it has been found that *CX3CR1*/*NF-κB* is involved in atherosclerosis and promotes monocyte adhesion and cell migration [38]. As a key gene in this study, *CX3CR1* may exert its effect on carotid plaques through *NF-κB*. The *NF-κB* pathway also interacts with the TLR signaling pathway. It has been found that *TLR4* mediates the activation of the *NF-κB* pathway and contributes to the development of heart failure by inducing oxidative stress and inflammation, impairing endothelial cells, increasing cardiac fibrosis, and promoting cardiomyocyte hypertrophy, apoptosis, pyroptosis, and autophagy, thereby causing damage to cardiomyocytes [39]. The study found that the *TLR4/NF-κB* pathway prevents ischemic reperfusion injury by regulating the polarization phenotype of macrophages towards M1 macrophages [40]. The TLR and *NF-κB* pathways can modulate immune cells and alleviate atherosclerosis, which improves the current understanding of the mechanism of macrophage modulation in carotid plaques.

This study also had some limitations. First, the amount of data and information related to carotid artery plaques in the public platforms was relatively limited. In addition, due to the differences in sample sources, collection, and data processing methods, the use of independent datasets for verification may lead to biased results. Moreover, the mechanisms through which the six key genes regulated the *NF-κB*/TLR pathway still remained unclear. In order to enhance the reliability of the current results, we plan to perform targeted experiments for further verification. For instance, animal models will be developed to simulate the formation process of human carotid artery plaques to more accurately evaluate the impact of the key genes. For clinical experiments, we intend to expand the sample size, covering patients from different geographical regions, age groups, and living environment backgrounds. By collecting and analyzing the clinical data of patients, such as medical history, symptoms, physical examination results, and various laboratory test data, we will be able to comprehensively explore the relationship between carotid artery plaques and the six key genes.

## 5. Conclusion

To conclude, this study comprehensively analyzed the single-cell transcriptomic data from patients with carotid plaques to reveal the regulatory role of macrophage subpopulations in carotid plaque formation during atherosclerosis. Six key genes associated with carotid plaque and macrophage regulation were discovered, and their accurate diagnostic performance was validated. In addition, these genes had potent regulatory effects on inflammation-related signaling pathways, such as TLR and *NF-κB* signaling pathways, and mediated multiple cell death types. However, some limitations in the present study should be equally noted. The data analyzed in this work were mainly collected from public databases and lacked experimental samples. Thus, follow-up experiments on tissue samples are needed to verify the regulatory mechanisms of inflammation-related pathways and macrophage-related marker genes in carotid plaque formation.

## Data Availability

The datasets generated and/or analyzed during the current study are available in the GSE120521 repository;https://www.ncbi.nlm.nih.gov/geo/query/acc.cgi?acc= GSE120521, GSE41571 repository;https://www.ncbi.nlm.nih.gov/geo/query/acc.cgi?acc= GSE41571, and GSE253903 repository;https://www.ncbi.nlm.nih.gov/geo/query/acc.cgi?acc= GSE253903.

## Nomenclature

WGCNA: Weighted gene correlation network analysis
TLR: Toll-like receptor
TME: Tumor microenvironment
TOM: Topology overlap matrix
PCA: Principal components analysis
GEO: Gene Expression Omnibus
tSNE: t-Distributed stochastic neighbor embedding
scRNA: Single-cell RNA
NES: Normalized enrichment score
TAM: Tumor-associated macrophage
AGE: Advanced glycation end product
ssGSEA: Single-sample gene set enrichment analysis
VSMC: Vascular smooth muscle cell.

## Supporting Information

Supporting Information 1 Table S1. The primer sequences in this study.

Supporting Information 2 Table S2. Statistics on the proportion of each cell subpopulation in symptomatic and asymptomatic samples of carotid plaques.

Supporting Information 3 Figure S1. The results of WGCNA. (A) Sample clustering tree. (B,C) Soft threshold screening for carotid plaque sample clustering by WGCNA.

Supporting Information 4 Table S3. The phenotypes of the samples.
