# Supplementary material for: Single-Cell Data and Weighted Correlation Network Analysis Revealed the Regulatory Mechanisms of Macrophages in Carotid Plaques
Source: J Immunol Res. 2025 Jul 21;2025:9987367. doi: 10.1155/jimr/9987367 (PMC12303652; doi:10.1155/jimr/9987367)
Supplement: Supporting Information 1 — Table S1. The primer sequences in this study. [file 9987367.f1.docx]

| Genes | Forward | Reverse |
| --- | --- | --- |
| *CX3CR1* | GAGCATCACTGACATCTACCTCC | AGAAGGCAGTCGTGAGCTTGCA |
| *ADPGK* | ATGGAGGGACAAAGCAAGGAGC | GCTCCCTGTTAGTCATACTGGC |
| *ATP6V1F* | GGGAGCTTAACAAGAACCGCCA | CTGCGATGTACTGGTTGATGAGG |
| *MYO9B* | ACTTCACGGAGCAGTTCCAGGT | TGACGCTCTGTCTCCTTCAGGA |
| *RNF135* | TGCTGCAAGACCTGGTGGACAA | CAAGCTGTCTCACCATGTCAGTC |
| *SLC7A8* | GCATACGTCACTGCAATGTCCC | GGAGCCATTGACTCCACCAAAC |
| GAPDH | GTCTCCTCTGACTTCAACAGCG | ACCACCCTGTTGCTGTAGCCAA |

Table S1. The primer sequences in this study.
