# Supplementary material for: Single-Cell Data and Weighted Correlation Network Analysis Revealed the Regulatory Mechanisms of Macrophages in Carotid Plaques
Source: J Immunol Res. 2025 Jul 21;2025:9987367. doi: 10.1155/jimr/9987367 (PMC12303652; doi:10.1155/jimr/9987367)
Supplement: Supporting Information 2 — Table S2. Statistics on the proportion of each cell subpopulation in symptomatic and asymptomatic samples of carotid plaques. [file 9987367.f2.docx]

| Cell types | Asymptomatic | Symptomatic |
| --- | --- | --- |
| B cells | 1.00% | 1.00% |
| Cycling cells | 2.00% | 1.00% |
| Cytotoxic T cells | 28.00% | 27.00% |
| Endothelial cells | 9.00% | 6.00% |
| Macrophages | 23.00% | 24.00% |
| Mast cells | 1.00% | 2.00% |
| Monocyte | 6.00% | 7.00% |
| Neutrophil | 2.00% | 1.00% |
| NKT cells | 2.00% | 2.00% |
| Plasma cells | 1.00% | 4.00% |
| Plasmacytoid dendritic cell (pDC) | 0.00% | 2.00% |
| Smooth muscle cell 1 | 23.00% | 21.00% |
| Smooth muscle cell 2 | 2.00% | 1.00% |

Table S2. Statistics on the proportion of each cell subpopulation in symptomatic and asymptomatic samples of carotid plaques
