# Supplementary figures and images for: Single-Cell Data and Weighted Correlation Network Analysis Revealed the Regulatory Mechanisms of Macrophages in Carotid Plaques
Source: J Immunol Res. 2025 Jul 21;2025:9987367. doi: 10.1155/jimr/9987367 (PMC12303652; doi:10.1155/jimr/9987367)

A

## Sample clustering

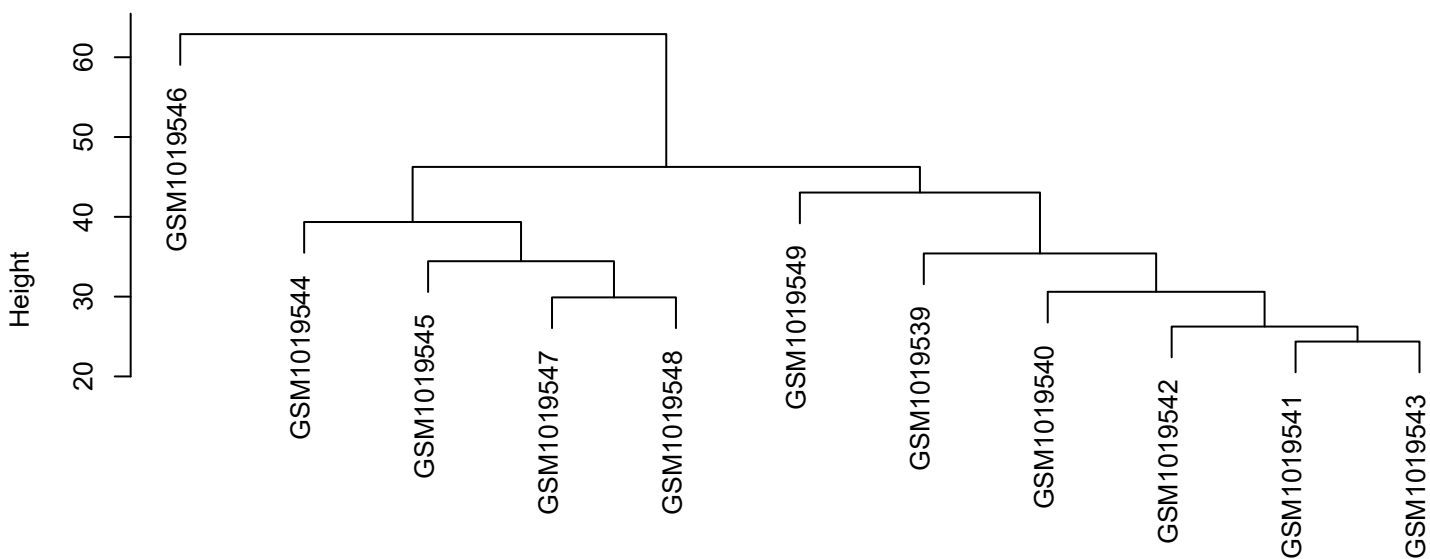

B

## Scale independence

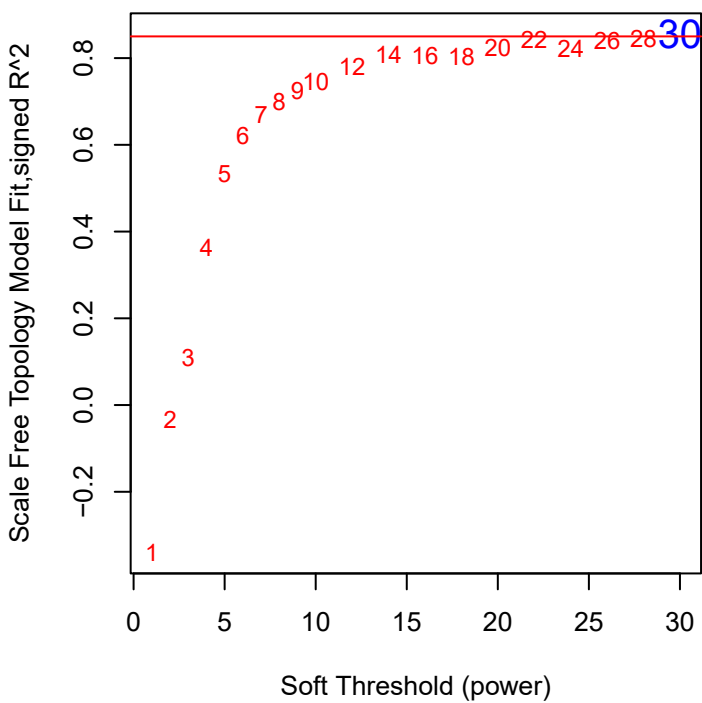

C

## Mean connectivity

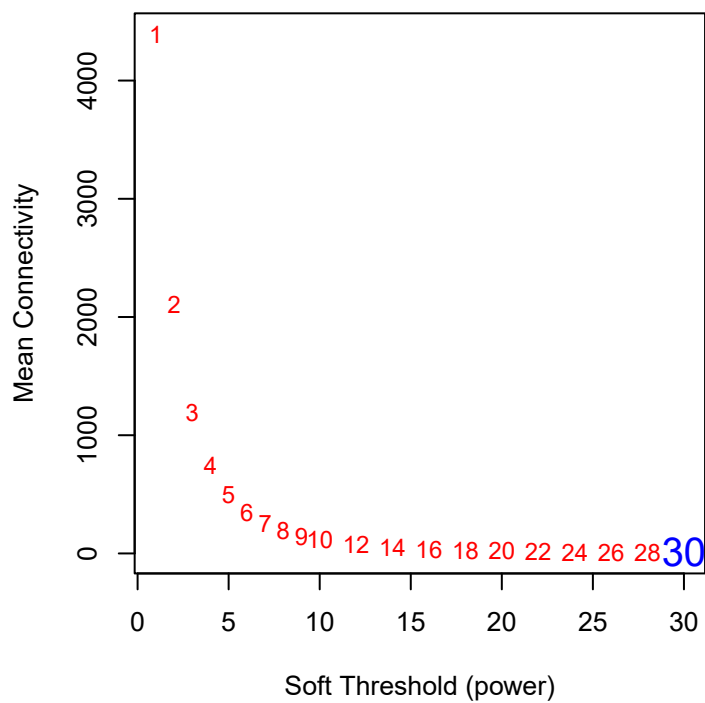

Supplement: Supporting Information 3 — Figure S1. The results of WGCNA. (A) Sample clustering tree. (B,C) Soft threshold screening for carotid plaque sample clustering by WGCNA. [file 9987367.f3.pdf]
