# Supplementary material for: Single-Cell Data and Weighted Correlation Network Analysis Revealed the Regulatory Mechanisms of Macrophages in Carotid Plaques
Source: J Immunol Res. 2025 Jul 21;2025:9987367. doi: 10.1155/jimr/9987367 (PMC12303652; doi:10.1155/jimr/9987367)
Supplement: Supporting Information 4 — Table S3. The phenotypes of the samples. [file 9987367.f4.docx]

Table S3. The phenotypes of the samples.

| Samples | Title |
| --- | --- |
| [GSM1019539](https://www.ncbi.nlm.nih.gov/geo/query/acc.cgi?acc=GSM1019539) | Ruptured Plaque, biological rep 1 |
| [GSM1019540](https://www.ncbi.nlm.nih.gov/geo/query/acc.cgi?acc=GSM1019540) | Ruptured Plaque, biological rep 2 |
| [GSM1019541](https://www.ncbi.nlm.nih.gov/geo/query/acc.cgi?acc=GSM1019541) | Ruptured Plaque, biological rep 3 |
| [GSM1019542](https://www.ncbi.nlm.nih.gov/geo/query/acc.cgi?acc=GSM1019542) | Ruptured Plaque, biological rep 4 |
| [GSM1019543](https://www.ncbi.nlm.nih.gov/geo/query/acc.cgi?acc=GSM1019543) | Ruptured Plaque, biological rep 5 |
| [GSM1019544](https://www.ncbi.nlm.nih.gov/geo/query/acc.cgi?acc=GSM1019544) | Stable Plaque, biological rep 1 |
| [GSM1019545](https://www.ncbi.nlm.nih.gov/geo/query/acc.cgi?acc=GSM1019545) | Stable Plaque, biological rep 2 |
| [GSM1019546](https://www.ncbi.nlm.nih.gov/geo/query/acc.cgi?acc=GSM1019546) | Stable Plaque, biological rep 3 |
| [GSM1019547](https://www.ncbi.nlm.nih.gov/geo/query/acc.cgi?acc=GSM1019547) | Stable Plaque, biological rep 4 |
| [GSM1019548](https://www.ncbi.nlm.nih.gov/geo/query/acc.cgi?acc=GSM1019548) | Stable Plaque, biological rep 5 |
| [GSM1019549](https://www.ncbi.nlm.nih.gov/geo/query/acc.cgi?acc=GSM1019549) | Stable Plaque, biological rep 6 |
